# Impact of admission triglyceride for early outcome in diabetic patients with stable coronary artery disease

**DOI:** 10.1186/1476-511X-13-73

**Published:** 2014-04-27

**Authors:** Xiao-Lin Li, Li-Feng Hong, Song-Hui Luo, Yuan-Lin Guo, Cheng-Gang Zhu, Jing Sun, Qian Dong, Ping Qing, Rui-Xia Xu, Jun Liu, Sha Li, Na-Qiong Wu, Geng Liu, Jian-Jun Li

**Affiliations:** 1 Division of Dyslipidemia, State Key Laboratory of Cardiovascular Disease, Fu Wai Hospital, National Center for Cardiovascular Diseases, Chinese Academy of Medical Sciences and Peking Union Medical College, Beijing 100037, China; 2 Division of Cardiology, Guangci Hospital affiliated Medical College of Wuhan University & the Fifth Hospital of Wuhan, Wuhan 430050, China

**Keywords:** Triglyceride, Hemoglobin A1C, Stable angina pectoris, Coronary artery disease, Outcome

## Abstract

**Background:**

The role of triglyceride (TG) in predicting the outcomes in diabetic patients with coronary artery disease (CAD) has not been well investigated.

**Methods:**

A total of 329 cases with stable angina pectoris (SAP) were prospectively enrolled and followed up for an average of 12 months. They were classified into the two groups according to the cut-off values of predicting early outcome of fasting TG level (low group <1.2 mmol/L, n = 103; High group ≥1.2 mmol/L, n = 226). The relationship between the TG levels and early outcomes were evaluated.

**Results:**

High TG group showed severer lipid profile and elevated inflammatory markers. During an average of 12-month follow-up, 47 out of 329 patients suffered from pre-specified outcomes. Area under the receivers operating characteristic curve suggested that TG, similar to serum *Hemoglobin A1C* (HbA1C), was a significant predictor of early outcome for diabetic patients with SAP (P = 0.002). In Cox regression models, after adjusted age, gender, body mass index, other lipid parameters, fasting blood glucose, high sensitivity C-reactive protein, neutrophil count and HbA1C, TG remained as an independent predictor of adverse prognosis.

**Conclusions:**

High level of fasting TG (≥1.2 mmol/L) was an independent predictor for early outcome of diabetic patients with SAP as like as HBA1c and number of affected coronary arteries in the era of revascularization and statin therapeutics.

## Introduction

Dyslipidemia is a hitherto well-known and pivotal risk factor for development of coronary artery disease (CAD). High levels of fasting triglyceride (TG) and low levels of high-density lipoprotein cholesterol (HDL-C) has long been regarded as potently atherogenic dyslipidemia in patients with and without diabetes mellitus (DM) [1-5]. Meanwhile, it has been demonstrated that concurrence of high fasting TG levels and low HDL-C levels, generally expressed as index of atherogenic dyslipidemia, was strongly associated with insulin resistance or glucose metabolic intolerance and high vulnerable to CAD [5-7]. In fact, several prospective epidemiological studies have reported that there are positive relationship between plasma TG concentration and the risk of CAD, especially from date of univariate analysis. However, this association disappeared after adjusting for HDL-C, low-density lipoprotein cholesterol (LDL-C) or non-HDL-C by using the multivariate regression models [8-10].

Meanwhile, the majority of evidence for elevated TG level as an independent risk for CAD derived from trials on primary prevention [10]. Several investigations on the association of high levels of fasting TG between CAD and its mortality has led to conflict results, especially in different ethnic backgrounds of the secondary prevention [6,11-17]. Moreover, whether the values of fasting TG should be tailored at specified point for diabetic population are largely unknown, as it may exhibit a more significantly detrimental role even when present at relatively “normal” levels while synergized with other dyslipidemia, disorder of glucose metalism and inflammatory biomarkers such as c-reactive protein (CRP) [1,18-22]. In addition, although several studies has demonstrated the positive correlation of high TG/HDL-C index and severity of CAD but evidence regarding whether the high levels of admission fasting TG could also provide any additional prognostic information for diabetic patients with stable CAD remained to be elucidated [20,23,24]. More importantly, the role of TG concentrations in predicting the clinical outcomes in the era of revascularization and statin therapeutics has currently not been established.

The aim of this study, therefore, was to prospectively investigate the predictive ability of baseline fasting TG levels for early outcome in Chinese Han diabetic patients with stable angina pectoris (SAP) underwent coronary angiography.

## Methods

### Patients population and study protocol

From June 2011 through March 2012, we prospectively enrolled 329 consecutive patients (73.3% of males, aged form 34 to 82 years with an average age of 59.3 years) diagnosed with type 2 diabetic patients and typical stable exertional angina pectoris referred for selective coronary angiography at our center. Patients without significant CAD and with type 1 diabetes mellitus, ACS, significant hematologic disorders (white blood cell count ≤3.5×10^9^/L or ≥20×10^9^/L), infectious or inflammatory disease, severe liver and/or renal insufficiency were excluded from the current study. All subjects enrolled were underwent detailed clinical, hematologic and angiographic examination for assessment of the cardiac status and were asked for their present and past history about risk of traditional risk factors of CAD such as smoking habits, hypertension, hyperlipidemia, obesity, diabetes mellitus, previous stroke, peripheral vascular disease, family history of CAD and non-cardiovascular diseases.

Hypertension was defined as repeated blood pressure measurements ≥140/90 mmHg and was assumed to be present in patients taking anti-hypertensive drugs. Diabetes mellitus was diagnosed in patients with fasting serum glucose level of ≥6.99 mmol/L in multiple determinations or under active treatment with insulin or oral hypoglycemic agents. Hyperlipidemia was considered to be present in patients with fasting total cholesterol (TC) ≥5.2 mmol/L or TG ≥1.7 mmol/L. CAD was defined as the presence of significant obstructive stenosis, at least 50% of the vessel lumen diameters, in any of the main coronary arteries by at least two independent senior interventional cardiologists based on quantity coronary angiography (QCA). The severity of CAD was scored as 1 (single vessel disease), 2 (two-vessel disease), 3 (three-vessel disease and/or left main stem disease and/or equally affected of left anterior descending and left circumflex branch). Stent implantation, periprocedural medical treatment and care were performed according to standard criteria when there were indicative of revascularization. Postinterventional antiplatelet therapy consisted of clopidogrel and aspirin with formal dosage. Drug eluting stents were majorly implantation. The left ventricular ejection fraction was evaluated by echocardiograph using the area-length methods with modified Simpson’s rule.

The study complied with the Declaration of Helsinki, and was approved by the hospital ethnic review board (Fu Wai Hospital & National Center for Cardiovascular Diseases, Beijing, China). Informed written consent was obtained from all patients included in this analysis.

### Follow up and study endpoints

The follow up protocol after discharge consisted of a phone or clinic interview. Patients were followed up for an average of 12 months. The pre-specified clinical end points were defined as cardiac causative death, nonfatal MI, revascularization, and re-hospitalization due to attack of acute coronary syndrome.

### Measurements of biomarkers

Venous blood samples were obtained from each patient at baseline upon admission. TC and TG were measured by enzymatic methods and HDL-C by a direct method (Roche Diagnostics, Basel, Switzerland). LDL-C was obtained by Friedewald’s formula (if fasting triglycerides < 3.39 mmol/l) or by ultracentrifugation. ApoB was measured by an immunoturbidimetric method (Tina-quant, Roche Diagnostics) calibrated against the World Health Organization/International Federation of Clinical Chemistry reference standard SP3–07. The levels of hemoglobin A1c (HbA1c) were measured using the Tosoh G7 Automate HPLC Analyzer (TOSOH Bioscience, Japan). The TG/HDL-C index was calculated from ratio of fasting TG to HDL-C ratio. Non-HDL cholesterol was calculated by subtracting HDL cholesterol from total cholesterol. LDL-C/HDL-C ratio expressed the ratio of LDL-C and HDL-C. The levels of high-sensitivity-CRP were determined using immunoturbidimetry (Beckmann Assay 360, Bera, Calif., USA) according to our previously reported. The median normal value for hs-CRP is 0.8 mg/L, with 90% of normal values <0.3 mg/L, and a lower detection limit of 0.2 mg/L. The inter-assay and intra-assay coefficients of variation were <5%, respectively. All other included biomarkers were analyzed by standard hematological and biochemical tests.

### Statistical analysis

Quantitative variables were expressed as mean ± standard deviation (SD), and qualitative variables were expressed as numbers and percentages. Continuous variables and categorical variables were analyzed by the two group’s *t* test, or chi-squared statistic tests when appropriate. Receivers operating characteristic (ROC) curves were constructed at the most discriminating cutoff point values aimed to document the predictive power of fasting triglycerides and other biomarkers for early outcome in the study population. Based on the cutoff values of admission fasting TG, the enrolled patients were classified into two groups (low group < 1.2 mmol/L, n = 103; High group ≥ 1.2 mmol/L, n = 226). Predictive effect of fasting TG and other biomarkers for 12-month outcomes was carried by two multivariate Cox proportional hazard models using forward stepwise selection process: model one used crude values of fasting TG and HbA1c; model two used tertiles of fasting TG and HbA1c. Event-free survival curves were constructed using the Kaplan-Meier methods and compared using log-rank test. A p value of less than 0.05 was considered as statistically significant. Statistical studies were carried out with the SPSS program (version 19.0, SPSS, Chicago, Illinois, USA).

## Results

### Baseline characteristics

**Figure 1:**
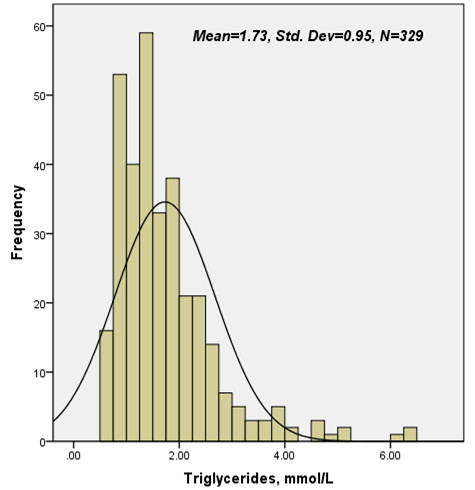
Distribution of admission fasting triglycerides in the study population.

The study population of current observation consisted of 329 diabetic patients underwent coronary angiography with an average follow-up time of 12 months (ranged from 20 to 448 days). The baseline characteristics and laboratory findings of the enrolled subjects by fasting TG distribution (Figure 1) and cut-off values of predictive early outcome in the study population (low group <1.2 mmol/L, n = 103; high group ≥1.2 mmol/L, n = 236) were summarized in Table 1 and Table 2. In brief, patients with higher TG levels were middle aged, higher body mass index (BMI) and accompanied with various other dyslipidemia and abnormal fasting blood glucose. Meanwhile, the major inflammatory and oxidative stress biomarkers such as leucocytes count, uric acid between the two groups showed significant unbalanced. However, levels of high sensitivity CRP, fibrinogen, D-dimer, HbA1c and other clinical characteristics such as involved numbers of affected coronary artery, cases of underwent secondary prevention and/or drug eluting stent implantation were well matched.

**Table 1:** Baseline demographic, clinical characteristics according to cut-off value of fasting triglycerides in the study population

**Variables**	**Total**	** Fasting triglycerides**		**P-value**
		**<1.2 mmol/L (N = 103)**	**≥1.2 mmol/L (N = 226)**	
Demographics				
Age, years	59.3 ± 9.3	60.9 ± 9.6	58.7 ± 9.1	0.043
Male gender	241 (73.3)	77 (74.8)	164 (72.6)	0.677
Body mass index	25.5 ± 3.0	24.9 ± 3.0	25.7 ± 3.0	0.045
Risk factors				
Current Smoking	181 (55.0)	59 (57.3)	122 (54.0)	0.577
Hypertension	221 (62.1)	64 (62.1)	157 (69.5)	0.189
Hyperlipidemia	263 (79.9)	76 (73.8)	187 (82.7)	0.060
Peripheral vascular disease	6 (1.8)	2 (1.9)	4 (1.8)	0.914
Prior Stroke	13 (4.0)	4 (3.9)	9 (4.0)	0.966
Family history of CAD	36 (10.9)	10 (9.7)	26 (11.5)	0.628
Angiographic findings				0.481
1-vessel disease	96 (29.2)	30 (29.1)	66 (29.2)	
2-vessel disease	80 (24.3)	21 (20.4)	59 (26.1)	
3-vessel disease	153 (46.5)	52 (50.5)	101 (44.7)	
LVEF (%)	62.0 ± 8.3	61.8 ± 8.8	62.1 ± 8.1	0.749
Medical treatment				
Aspirin	321 (97.6)	100 (97.1)	221 (97.8)	0.702
Clopidogrel	315 (95.7)	100 (97.1)	215 (95.1)	0.415
Beta-blocker	271 (82.7)	79 (76.7)	193 (85.4)	0.053
ACE-I	85 (25.8)	29 (28.2)	56 (24.8)	0.516
Statin	320 (97.3)	101 (98.1)	219 (96.9)	0.551
DES implantation	67 (20.4)	22 (21.4)	45 (19.9)	0.762

*CAD* Coronary artery disease, *LV-EF* Left ventricular ejection fraction, *ACE-I* Angiotensin converting enzyme inhibitors, *DES* Drug eluting stent.

**Table 2:** Baseline laboratory characteristics according to cut-off value of serum Triglycerides in the study population

**Variables**	**Total**	** Fasting triglycerides**		**P-value**
		**<1.2 mmol/L (N = 103)**	**≥2 mmol/L (N = 226)**	
Biochemical markers				
hs-CRP (mg/L)	3.2 ± 4.1	3.5 ± 4.5	3.1 ± 3.9	0.440
Leucocyte count (10^9^/L)	6.5 ± 1.6	6.2 ± 1.5	6.6 ± 1.6	0.048
Neutrophil count (10^9^/L)	3.8 ± 1.2	3.6 ± 1.1	3.9 ± 1.2	0.041
Neutrophil/lymphocyte ratio	2.2 ± 1.1	2.1 ± 1.0	2.2 ± 1.1	0.530
Fasting blood glucose (mmol/L)	6.1 ± 2.2	5.7 ± 1.9	6.3 ± 2.3	0.025
Hemoglobin A1C (%)	6.9 ± 1.4	6.7 ± 1.3	6.9 ± 1.4	0.149
D-dimer (ug/mL)	0.4 ± 0.5	0.4 ± 0.6	0.4 ± 0.4	0.252
Fibrinogen (g/L)	3.1 ± 0.8	3.1 ± 0.8	3.1 ± 0.9	0.644
Endothelin-1 (fmol/ml)	0.6 ± 0.3	0.6 ± 0.3	0.6 ± 0.3	0.587
Alkaline phosphatase (IU/L)	63.5 ± 18.6	62.0 ± 20.1	64.2 ± 17.9	0.318
Uric acid (mmol/L)	338.3 ± 78.9	314.9 ± 75.9	348.9 ± 78.1	0.000
Creatinine (umol/L)	76.3 ± 15.6	74. 4 ± 14.0	77.2 ± 16.2	0.138
Lipid profile				
Total cholesterol (mmol/L)	4.0 ± 1.0	3.5 ± 0.8	4.2 ± 1.1	0.000
LDL-C (mmol/L)	2.4 ± 0.9	2.1 ± 0.7	2.5 ± 0.9	0.000
HDL-C (mmol/L)	1.1 ± 0.3	1.1 ± 0.3	1.0 ± 0.3	0.002
Lipoprotein (a) (mg/L)	244.4 ± 244.5	267.9 ± 218.9	233.6 ± 254.9	0.237
apoA(g/L)	1.4 ± 0.3	1.4 ± 0.3	1.4 ± 0.3	0.477
apoB(g/L)	1.0 ± 0.3	0.9 ± 0.2	1.1 ± 0.3	0.000
TG/HDL-C index	1.7 ± 1.3	0.8 ± 0.3	2.2 ± 1.3	0.000
Non-HDL-C (mmol/L)	2.9 ± 0.9	2.4 ± 0.7	3.1 ± 0.9	0.000
LDL-C/HDL-C ratio	2.3 ± 0.8	1.9 ± 0.7	2.4 ± 0.8	0.000

*hs-CRP* high sensitivity C-reactive protein, *LDL-C* Low density lipoprotein cholesterol, *HDL-C* High density lipoprotein cholesterol, *Non-HDL-C* Non-High density lipoprotein cholesterol = Total cholesterol subtracts to high density lipoprotein cholesterol, *TG/HDL-C index* Triglycerides/high density lipoprotein cholesterol ratio, *LDL-C/HDL-C ratio* Low density lipoprotein cholesterol/High density lipoprotein cholesterol ratio.

### Utility of TG for predicting early outcomes

**Figure 2:**
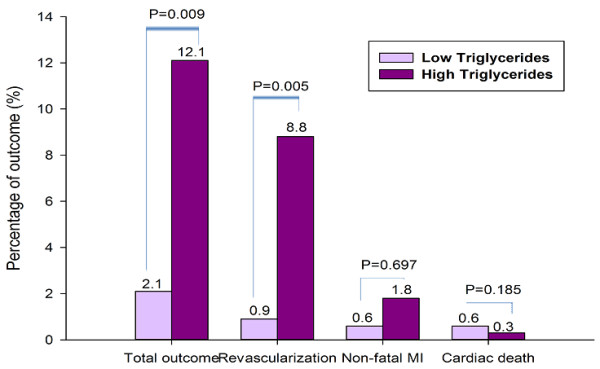
Correlation of admission fasting triglycerides and 12-month outcome.

**Figure 3:**
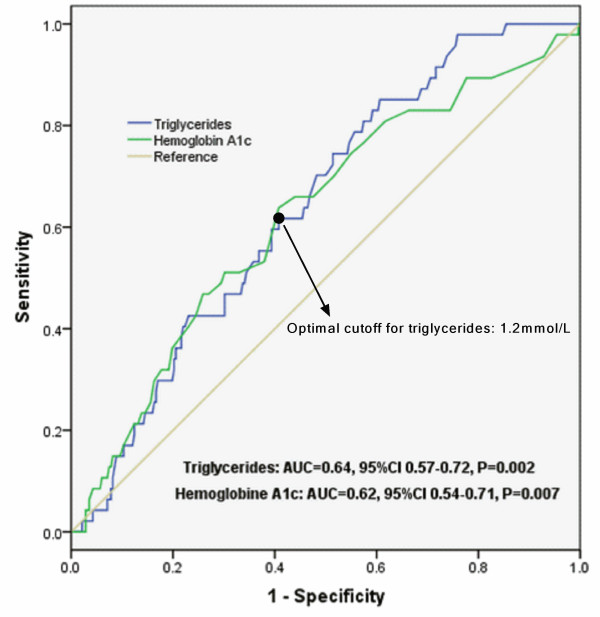
ROC curves showed discriminatory power of baseline serum Triglycerides and hemoglobin A1c on early outcome of the study population.

During an average 12-month follow-up, 47 out of the 329 patients underwent adverse outcome (Figure 2). There were significant associations among baseline TG levels and incidence of total outcome, revascularization (P = 0.009 and 0.005, respectively), but not for nonfatal MI or cardiac death (P = 0.697 and 0.185, respectively) during the follow-up period. Area under the receivers operating characteristic (ROC) curves (Figure 3 and Table 3) suggested that baseline TG, beyond other lipid parameters and nonspecific inflammatory biomarkers, was a significant predictor for early outcome of diabetic patients with SAP (AUC = 0.64, 95% CI 0.57-0.72, P = 0.002).

**Table 3:** Comparison of AUC among lipid profiles, glucose intolerance parameters and inflammatory biomarkers for predicted early outcome in the study population

**Variables**	**AUC**	**95% CI**	**P-value**
Lipid profiles			
Fasting triglycerides	0.64	0.57-0.72	0.002
TG/HDL-C index	0.62	0.54-0.69	0.012
ApoB	0.58	0.49-0.67	0.076
Non-HDL-C	0.56	0.46-0.65	0.230
LDL-C/HDL-C ratio	0.55	0.46-0.64	0.252
ApoA	0.54	0.45-0.64	0.363
Total cholesterol	0.53	0.44-0.63	0.470
LDL-C	0.53	0.44-0.63	0.495
HDL-C	0.49	0.40-0.59	0.935
Parameters of glucose intolerance			
Hemoglobin A1C	0.62	0.54-0.71	0.007
Fasting blood glucose	0.61	0.52-0.70	0.013
Inflammatory biomarkers			
hs-CRP	0.55	0.46-0.63	0.293
Neutrophil count	0.53	0.44-0.61	0.557

*TG/HDL-C index* Triglycerides/high density lipoprotein cholesterol index, *Non-HDL-C* Non-High density lipoprotein cholesterol = Total cholesterol subtracts to high density lipoprotein cholesterol, *LDL-C/HDL-C ratio* Low density lipoprotein cholesterol/High density lipoprotein cholesterol ratio, *LDL-C* Low density lipoprotein cholesterol, *HDL-C* High density lipoprotein cholesterol, *hs-CRP* high sensitivity C-reactive protein.

### Multivariate Cox proportional regression model and Kaplan- Meier curves of TG for predicting early outcomes

Multivariate analysis by Cox regression models (Table 4, model one using absolute values of admission fasting TG and model two using tertiles of fasting TG that controlled for major potential confounders (including age, gender, BMI, traditional cardiovascular risk factors, other lipid parameters and hematological index of differential at baseline) suggested that, apart from numbers of affected coronary arteries and serum HbA1C, fasting TG was remained as an independent predictor for overall outcome in patients with SAP (model one: HR = 1.29, 95% CI 1.01-3.93, P = 0.048; model two: HR = 1.53, 95%CI 1.06-2.21, P = 0.025, respectively).

**Table 4:** Cox proportional regression of Multivariate adjusted for independent predictors of 12-month total outcome

**Variables**	**HR**	**95% CI**	**P-value**
*Model 1*			
Triglycerides	1.29	1.01-3.93	0.048
Hemoglobin A1C	1.34	1.12-1.45	0.001
Numbers of affected coronary arteries	1.69	1.19-1.69	0.009
*Model 2*			
First tertile of triglycerde (reference)	1.00		
Second tertile of triglycerde	2.75	1.15-6.60	0.023
Third tertile of triglyceride	3.13	1.34-7.32	0.008
Numbers of affected coronary arteries	1.59	1.16-2.20	0.004

**Figure 4:**
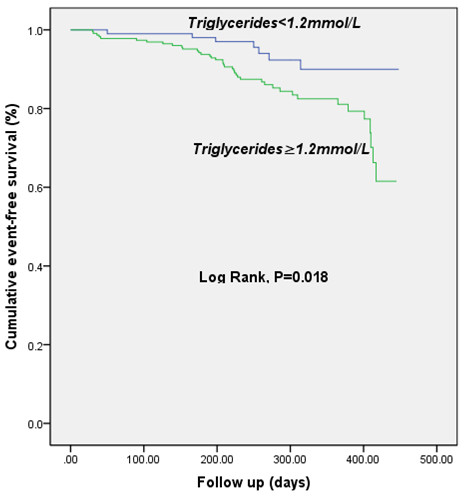
Kaplan-Meier curve for 12-month cumulative event-free survival based on cutoff value of baseline serum Triglycerides.

Kaplan-Meier curves for cumulative event-free survival based on cutoff values of admission fasting triglycerides were showed in Figure 4. High levels of serum fasting TG (≥1.2 mmol/L) was associated with increased early adverse outcome (Figure 4).

## Discussion

As far as our knowledge, this was the first study among Chinese type 2 diabetes with stable CAD to demonstrate that fasting TG on admission was an useful predictor for adverse outcomes independent of other traditional prognostic variables in the era of revascularization and statin therapy. The main findings of the present study are threefold. First, according to baseline characteristics of the current study, patients with higher fasting TG levels (≥1.2 mmol/L) were more prominent at groups of middle aged and higher BMI. Moreover, patients at high TG groups prone to be accompanied with other various dyslipidemia, impaired fasting glucose, high levels of inflammatory and oxidative response biomarkers such as leucocyte count, neutrophil count and uric acid. Second, in agreement with previous studies, as showed in ROC curves and bar graphs, our data further demonstrated that elevated fasting TG levels might be conferred to a useful discriminator for the presence of adverse events in diabetic patients with SAP. Third, both chi-squared for trend and multivariate Cox proportional regression analysis after adjusted major potential confounders were consistently indicated that fasting TG could provide with prognostic information in diabetic population with stable CAD and remained as an independent predictor for early outcome. Kaplan-Meier curve for cumulative event-free survival indicated that the high levels of TG were associated with increased adverse prognosis although the rate of statin administration and stent implantation were not different between the groups. Apparently, the present study not only confirmed the previous studies but also provided the novel information concerning the role of TG in predicting early outcomes in diabetic patients with stable CAD, especially in the era of revascularization and statin therapy.

Several lines of evidence have revealed a positive relation between TG and CAD and clinical outcomes in patients with or without diabetes owing to the role of TG-rich lipoproteins in atherothrombosis. The Copenhagen Male Study, which followed 2,906 white men over 8 years, demonstrated that fasting TG was independently associated with the incidence of CAD [25]. Data from Multiple Risk Factor Intervention Trial confirmed that either non-fasting or fasting TG is an independent risk factor for CAD [26]. Several mata-analyses showed that high concentration of TG was an independent risk factor for the morbidity and mortality rates of CAD in primary prevention [8-10]. Moreover, the Action to Control Cardiovascular Risk in Diabetes (ACCORD) study detected that combination therapy using lipid-lowering drugs for regulating both LDL-C and TG could lead to a significant benefit in patients with metabolic syndrome but not to those without [4]. Recently, Kasai et al. observed 1836 patients who underwent complete revascularization between 1984 and 1992, and evaluated the association between fasting TG level and all-cause and cardiac mortality for a median follow-up of 10.5-year period [16]. They found that elevated fasting TG levels were associated with increased risk of cardiac death after complete coronary revascularization. However, they did not demonstrated that there were significant associations of age, gender, presence of diabetes, levels of TC and HDL-C, the use of statins with all-cause and cardiac death in their subgroup analysis.

The disparities of our data from their study were summarized as followings: firstly, their patients were enrolled between 1984 and 1992 duration in which optimal pharmacological therapy for CAD was not widely performed clinically. Besides, prior cases were subjected to percutaneous coronary intervention (PCI) with simple balloon angioplasty, and no patients received stent implantation; Finally, the population received coronary artery bypass grafting (CABG) in previous studies were as higher as 32%. In current study, including subjects were confined at diabetic patients with an relatively short-term duration (from June 2011 through March 2012). Among studied population, majority of patients were received statin therapeutics, and their profiles of major lipid disorders were significantly improved such as perfect of targeted LDL-C. We affirmed that baseline fasting TG was a powerful predictor for early outcome in diabetic patients with stable CAD, similar to the evidence from American Heart Association which has recently suggested that the independent predictive values of TG levels as a causal factor in development of CAD remains as debatable [27]. Obviously, our study extended previous studies in that data provided the vital prognostic information regarding the role of fasting TG in diabetic subjects with stable CAD.

At the same time, our data suggested that patients with higher levels of admission fasting TG were more prone to receive revascularizations. The underlying hypothesis of current results might be consisted in three aspects. To begin with, high levels of fasting TG were not only implied with the severe disorder of glycolipid metabolism, but also low-grade of systematic inflammatory response, vascular dysfunction and potentially atherosclerotic progress in these settings [2,28-31]. Secondly, with the advent of statin therapeutic era, the directly pivotal role of LDL-C on the atherogenesis has been favorably controlled (an average of LDL-C = 2.4 ± 0.9 mmol/L in the studied population). Nonetheless, It has been reported that the residual risks of atherogenic dyslipidemia have been increasingly prominent and gradually emerged as a leading impeller of cardiovascular disease and its future event [32,33]. Therefore, the badly impacts of TG were not adjusted by multivariable analysis especially in the patients received of successfully complete revascularization and ideal glycemic control. Regarding to the current study population, all patients who had indications of revascularization were received complete intervention. Thirdly, several prior studies about secondary prevention of fasting TG had led to very similar results about the HR values (approximate to 1.5) [16,34]. Although those study population had different background of demographics and comorbidities, the reproducible of above result strikingly supported the consistent hypothesis that TG was an independent predictor of adverse outcome. Fourthly, epidemical cohorts about primary prevention also suggested a direct association between relatively high levels of TG and incidence of CAD and mortality [8-10]. Finally, our findings also supported the viewpoint that high levels of TG might be very probably to have potential impacts on the vasculature before the establishments of formal diagnosis for hypertriglyceridemia and led to fasting glucose impaired and/or overt atherosclerosis disease alone or coupled with other risk biomarkers [30,32,35].

Nonetheless, the limitations of our study are obvious. First of all, the sample scale of the current study was relatively small and enrolled patients were entirely Chinese Han population. Besides, the duration of follow-up period was comparably in short term and unavoidably led to the bias for totally observing the outcome. Furthermore, the main method of revascularization of present investigation was confined to drug eluting stent implantation and although it might be mostly analogous to the real world of China, it inevitably implied with high incidence of target vascular revascularization. Besides, as the universal utilization of statin therapeutics, it will be difficult to deploy a subset analysis on the effect of statin therapy. Finally, our investigation failed to compare the predictive values of fasting and non-fasting TG at these settings.

Taken together, although TG was a paradox marker all along, the results of our study clearly suggested that high level of fasting TG (≥1.2 mmol/L) was an independent indicator of early outcome for diabetic patients with SAP as like as HBA1c and number of affected coronary arteries. Whether should be set a strict target value of fasting TG specifically for diabetic population with stable CAD aimed to reverse the “atypical atherogenic dyslipidemia” need to be seriously considered.

## Abbreviations

TG: Triglyceride; CAD: Coronary artery disease; SAP: Stable angina pectoris.

## Competing interests

The authors declare that they have no competing interests.

## Authors’ contributions

LXL and HLF collected and analyzed the data, drafted the manuscript. LJJ conceived and designed this study, interpreted the data, and edited the manuscript. GYL, ZCG, SJ, DQ, QP, XRX, LJ, LS, WNQ, LG and JLX collected and interpreted the data, and revised the manuscript. All authors read and approved the final manuscript.
